# Development and validation of a prognostic prediction model for iron metabolism-related genes in patients with pancreatic adenocarcinoma

**DOI:** 10.3389/fgene.2022.1058062

**Published:** 2023-01-04

**Authors:** Wenhan Wei, Bin Cao, Dongchao Xu, Yusheng Liu, Xiaofeng Zhang, Yu Wang

**Affiliations:** ^1^ Department of Gastroenterology, Affiliated Hangzhou First People’s Hospital, Zhejiang University School of Medicine, Hangzhou, China; ^2^ China State Key Laboratory of CAD&CG, Zhejiang University, Hangzhou, China; ^3^ Department of Pharmacy, First Affiliated Hospital, Huzhou University, Huzhou, China; ^4^ Hangzhou Institute of Digestive Diseases, Hangzhou, China; ^5^ Key Laboratory of Integrated Traditional Chinese and Western Medicine for Biliary and Pancreatic Diseases of Zhejiang Province, Hangzhou, China

**Keywords:** pancreatic adenocarcinoma, biomarker, prognostic model, iron metabolism-related, q-PCR validation

## Abstract

**Background:** Pancreatic adenocarcinoma (PAAD) is one of the most aggressive tumors of the digestive tract, with low surgical resection rate and insensitivity to radiotherapy and chemotherapy. Existing evidence suggests that regulation of ferroptosis can induce PAAD cell death, inhibit tumor growth, and may synergistically improve the sensitivity of other antitumor drugs. However, there is little of systematic research on iron metabolism-related genes in PAAD. In this study, a risk-score system of PAAD iron metabolism-related genes was designed and tested, and verified to be robust.

**Materials and Methods:** The TCGA database was used to download 177 PAAD patients’ message RNA (mRNA) expression profiles and clinical characteristics. By identifying dysregulated iron metabolism-related genes between PAAD related tissues and adjacent normal tissues, univariate Cox proportional hazards regression and LASSO regression algorithm were used to establish prognostic risk-score system and construct nomogram to estimate the 1-, 2-, 3-year survival in PAAD patients. Finally, selected genes were validated by quantitative PCR (q-PCR).

**Results:** A 9-gene related to iron metabolism risk-score system of PAAD was constructed and validated. The clinicopathological characteristics of age, histologic grade, pathologic stage, T stage, residual tumor, and primary therapy outcome were all worse in patients with a higher risk-score. Further, immunohistochemistry results of *SLC2A1*, *MBOAT2*, *XDH*, *CTSE*, *MOCOS*, and *ATP6V0A4* confirmed that patients with higher expression are more malignant. Then, a nomogram with 9-gene risk score system as a separate clinical factor was utilized to foretell the 1-, 2-, 3-year overall survival rate of PAAD patients. Results of q-PCR showed that 8 of the 9 genes screened were significantly up-regulated in at least one PAAD cell line, and one gene was significantly down-regulated in three PAAD cell lines.

**Conclusion:** To conclude, we generated a nine-gene system linked to iron metabolism as an independent indicator for predicting PAAD prognosis, therefore presenting a possible prognostic biomarker and potential treatment targets for PAAD.

## Introduction

Pancreatic adenocarcinoma (PAAD) is a very lethal and aggressive malignant tumor of the pancreas with a dismal prognosis (Gupta et al., 2017). According to GLOBOCAN statistics from International Cancer Research Institute (IARC) in 2020, there were 495,773 new cases and 466,003 deaths of PAAD, accounting for 2.6% of all new cancer cases and 4.7% all cancer deaths in 2020 (Sung et al., 2021). The median overall survival (OS) duration of patients with PAAD is less than 6 months, and overall 5-year survival rate is less than 5% (Long et al., 2014). Risk factors, including smoking, alcohol abuse, chronic pancreatitis, and diabetes mellitus, have been identified to contribute to the carcinogenic effects of PAAD (Burkey et al., 2014). The major reasons for the poor prognosis of PAAD are that the early symptoms are not specific, the lack of early detection strategies, and effective clinical treatment methods. Most PAAD patients are diagnosed with advanced disease, which usually precludes complete resection to greatly reduce the odds of a favorable treatment outcome (Siegel et al., 2018).

Iron is an important component in the regulation of metabolic homeostasis, and iron-dependent enzymes use it to execute a variety of vital biological processes. It is principally implicated in processes like DNA synthesis, ATP generation, and oxygen transportation (Zhang et al., 2019). Control of iron metabolism is fundamental to almost all known life, meanwhile, iron metabolism is also considered indispensable for cancer development (Manz et al., 2016). Unlike normal cells, supply of iron is often rate-limiting for fast growing cancer cells and are accordingly more vulnerable to iron reduction. Evidence from previous studies suggested that tumor cells may raise intracellular iron levels *via* regulating the expression of the transferrin receptor, ferroportin, and ferritin expression (Jeong et al., 2015; Schonberg et al., 2015). Tumor cell multiplication, infiltration, and metastasis are aided by dysregulation of iron metabolism-related genes (Jung et al., 2019). The gathering of iron may cause breaks in DNA strands and tumorigenesis (Legendre and Garcion, 2015). Iron is also involved in a variety of cell death processes, including ferroptosis, an iron-dependent type of controlled cell death (Dixon and Stockwell, 2014). Sufficient oxidative damage and/or inactivation/depletion of preventative particles against oxidative damage induce ferroptosis. Ferroptosis has been identified in a variety of cancers, including PAAD, breast cancer, and hepatocellular carcinoma (Lu et al., 2017). Since tumor cells are really sensitive to ferroptosis, triggering ferroptosis may also have significant therapeutic potential for tumor cells (Chen et al., 2020). Iron-Responsive Element Binding Protein 1 and 2 (*IREB1 and IREB2*) are genes in the iron system of regulation that also moderate iron metabolism and moreover take a role in cancerous cells remodeling, which leads to malignant progression (Zhang et al., 2017). Epidemiological investigation of the NIH-AARP diet and health study cohort revealed that consuming heme iron from red meat increases pancreatic cancer risk (Taunk et al., 2016). Consistently, Gaur et al. investigated the relationship between Iron metabolism and risk of cancer in the Swedish Apolipoprotein Mortality Risk (AMORIS) study, and found a positive association between standardized serum (SI) iron or standardized total-iron binding capacity (TIBC) and Pancreatic cancer [HR per SD of SI 1.03 (95 % CI 0.89–1.20), and HR per SD of TIBC 1.12 (0.97–1.30)] (Gaur et al., 2013). Experimental studies of iron overload support that iron plays a direct and causal role in diabetes pathogenesis mediated both by β cell failure and insulin resistance. Sachelly et al. observed a significant association between the combined effects of common variants in the hepcidin-regulating iron metabolism gene pathway and PAAD (Sachelly et al., 2021). The signals contributing the most to the association were from the HJV, TFR2, TFR1, BMP6, and HAMP genes (Sachelly et al., 2021). Although researchers pay more and more attention to the relationship between iron metabolism-related genes and PAAD, the relevant research is still insufficient.

**FIGURE 1:**
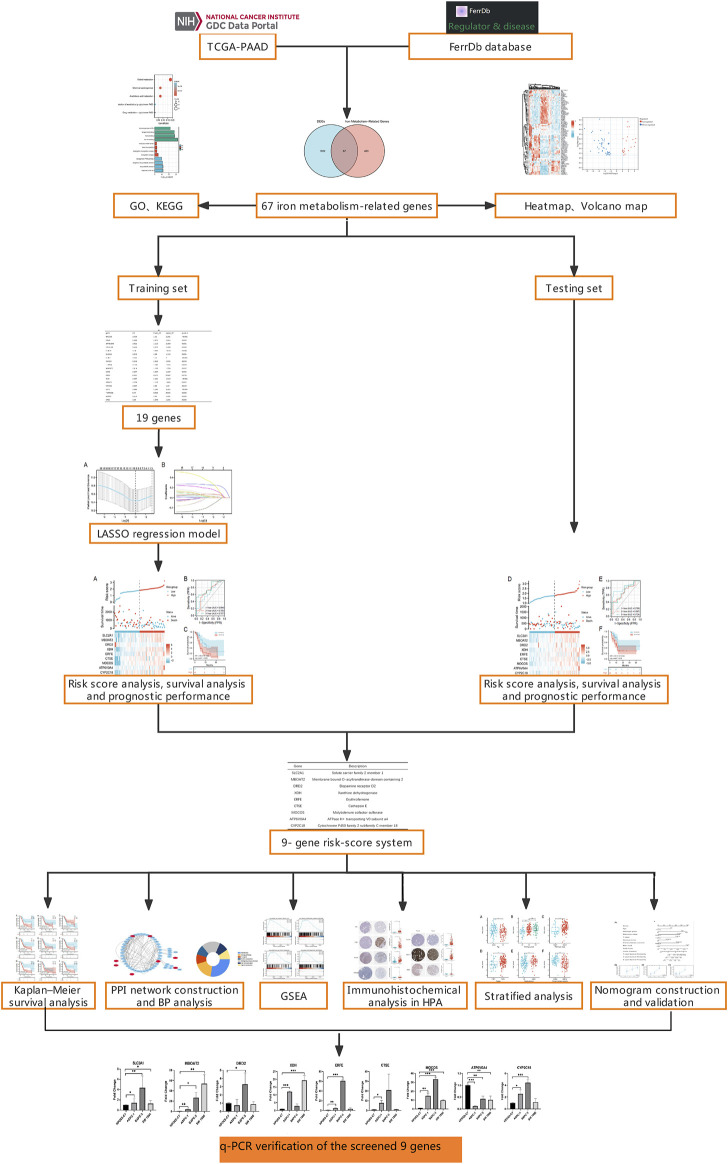
Research flow chart.

The purpose of this study was to create a predictive model for PAAD patients using iron metabolism-related genes. The Cancer Genome Atlas-Pancreatic Adenocarcinoma (TCGA-PAAD) database was used to get the mRNA expression profiles. Differentially expressed genes (DEGs) between PAAD-related tissues and normal tissues were identified using differential expression analysis. Then, DEGs related to iron metabolism were screened out, and comprehensive bioinformatics analyses were performed based on gene-expression levels. In addition, a 9-gene PAAD risk-score system were established by the Least Absolute Shrinkage and Selection Operator (LASSO) regression, and evaluated by risk score analysis, survival analysis and receiver operating characteristic (ROC) curves. Furthermore, we employed functional analysis and gene set enrichment analysis (GSEA) to evaluate potential iron metabolism pathways and processes in high-risk and low-risk populations. At the end of the study, we used a nomogram including age, sex, tumor TNM stage, histological grade, and a 9-gene risk scoring system as independent clinical components to predict 1-, 2-, and 3-year survival in PAAD patients. Figure 1 depicts the complete flow chart of the research.

## Methods

### Datasets and data processing

Gene expression data of PAAD samples were downloaded from the TCGA database (https://tcga-data.nci.nih.gov/tcga/). The PAAD RNA-seq data as well as clinical parameters were gathered from the TCGA portal, the clinical prognosis data supplemented from Liu’s literature (Liu et al., 2018). Gene expression microarray data was pre-processed and normalized by computed z-scores to have mean 0 and variance 1.

### Differential analysis

We analyzed DEGs between PAAD tumor tissues and normal adjacent tissues using the R software package “DESeq2” (version 3.6.3) (Robinson et al., 2010; McCarthy et al., 2012; Love et al., 2014; Ritchie et al., 2015). The DEGs were filtered by the criterion of *p <* 0.05 and absolute log2-fold change >1. The Molecular Signatures Database (MSigDB) version 7.1 was used to identify genes associated to iron metabolism (Subramanian et al., 2005; Liberzon et al., 2015). The overlapping genes between DEGs and iron metabolism-related genes were collected for further study.

### GO and KEGG pathway analysis

The unique biological properties of transcriptome and genomic data were determined utilizing Gene Ontology (GO) enrichment (Ashburner et al., 2000). For the characterization of pathways, complexes and networks, Kyoto Encyclopedia of Genes and Genomes (KEGG) pathway has been widely utilized (Richardson et al., 2019). To reveal the functional roles of DEGs, GO analysis [containing biological process (BP), molecular function (MF), and cellular component (CC)] and KEGG pathway analysis were performed with “clusterProfiler” package of R software using all present genes as background.

### Constructing and validating the risk-score system

To construct the iron metabolism-related genes risk score model, TCGA-PAAD samples were randomized divided into the training (50%) and testing set (50%). The training set was applied to train the risk score model, while the testing set was applied to evaluate its effectiveness. To ensure the robustness of our results, the random partitioning process was repeated ten times, and we present results as the mean of ten repeat simulations. To explore the correlation between expression level of overlapping genes and survival time, the univariate Cox analysis was carried out by the “survival” of R software to screen prognosis related genes (Wang et al., 2019). The statistically significant difference was defined as *p* < 0.05. To reduce the superabundance of prognostic genes of high dimension, the “glmnet” R package was employed to create a regression model by LASSO regression (Friedman et al., 2010). Next, using the LASSO regression coefficients of PAAD’s iron metabolism-related genes, a risk score model was created using the following formula.

$$Risk\;score=\overset{n}{\underset{i=1}{\sum }}\exp r_{genei}\times coefficient_{genei}$$

Further, the training set was also separated into high-risk and low-risk groups based on the median risk score value. The “survival” R package was employed to evaluate overall survival (OS) in both groups by Kaplan–Meier survival analysis. The “time ROC” R package was used to depict the distribution of ROC curves, and the areas under ROC curves (AUCs) were computed to verify the risk model’s efficiency. (Blanche et al., 2013).

### Explore the prognostic value and biological characteristics of screening genes in PAAD

We performed Kaplan–Meier survival analysis on the screened genes, and created a protein-protein interaction (PPI) network from the STRING database (http://string-db.org) with default parameters. We also used FunRich (version 3.1.3) for Biological Process (BP) analysis of the screened genes. The above-mentioned R packages were used to compute DEGs between high- and low-risk groups in the training data. Next, in comparison to the low-risk group, GSEA (http://software.broadinstitute.org/gsea/index.jsp) was used to determine the hallmarks of the high-risk group.

The identified mutations of the screened genes were analyzed at cBioPortal (www.cbioportal.org/). To explore the interaction between the screened genes and DEGs from tumor tissues of PAAD patient *vs* normal pancreatic tissues, we performed Spearman correlation analysis on the screened genes and the top 20 genes in log2-fold change absolute value.

The Human Protein Atlas (http://proteinatlas.org; HPA) was used to investigate the protein expression of indicated genes, as well as the analysis of the transcriptome expression level of each gene between normal and tumor. We also divided patients into subgroups based on clinicopathological characteristics, including age, histologic grade, pathologic stage, T stage, residual tumor, and primary therapy outcome. By using the “ggpubr” R package, we plotted boxplots to determine the relationship between risk scores and clinical characteristics.

### Development and evaluation of the nomogram

We employed univariate and multivariate Cox regression analysis on clinicopathological factors, such as age, histologic grade, pathologic stage, T stage, residual tumor, and primary therapy outcome, to see whether the risk score system could be used as an independent predictor or not. Furtherly, we designed a nomogram for predicting OS probabilities at 1-, 2- and 3-year by the ‘rms’ R package. The discriminative power of the nomogram was assessed via Harrell’s concordance index (C-index) and calibration plot (Alba et al., 2017).

### Verification of screened genes by quantitative real-time PCR

The whole RNA from pancreatic epithelial cells (HPDE6-C7) and pancreatic cancer cells (ASPC-1, BXPC-3, SW 1990) was extracted using an RNA Extraction Kit (Beyotime) and subsequently reverse transcribed into cDNA. Amplification reactions were performed with ABI7500 quantitative PCR system (Thermofisher) using UltraSYBR Mixture according to the manufacturer’s manual (Cwbio). The 2^−ΔΔ^CT approach was used to compute the relative expression levels of genes, which were measured in triplicate. GAPDH serves as a reference by which to compare the relative gene expression levels. Primers are listed in the Supplementary Table S2, which were designed by Primer Bank (https://pga.mgh.harvard.edu/primerbank/index.html).

### Statistical analysis

In this study, R software (version 3.6.3) and SPSS 20.0 software were used to conduct statistical analyses. For survival analysis, the Kaplan-Meier analysis and the log-rank test were employed. For correlation analysis, due to the non-normal distribution of the data, Spearman’s correlation test was performed. We considered the hazard ratio (HR) and confidence interval (CI) of 95% in this study to be appropriate and meaningful. For Real-time PCR, Student t-tests and Kruskal-Wallis tests were used to analyze continuous variables with normal and non-normal distribution respectively. In general, a *P*-value of less than 0.05 was judged statistical significance.

## Results

### Identification of iron metabolism-related gene in patients with PAAD

**FIGURE 2:**
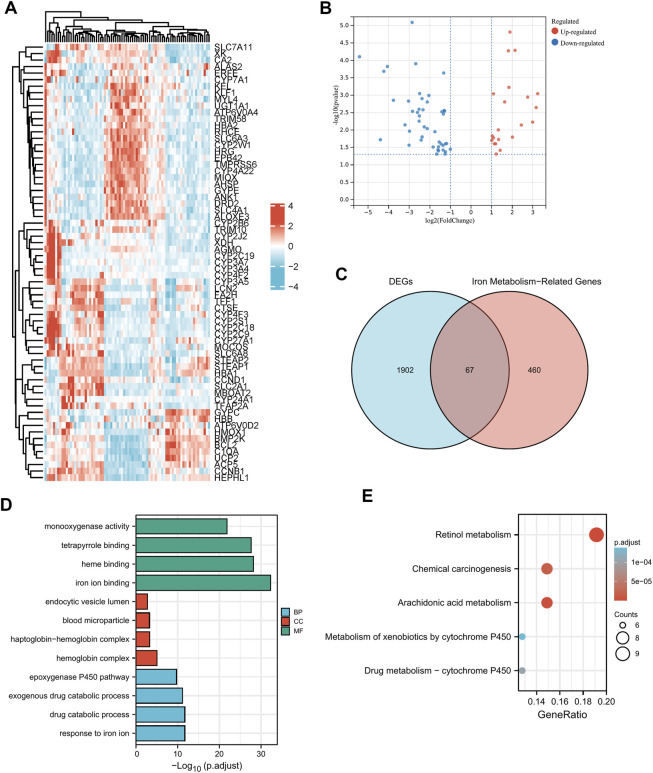
Functional enrichment analysis of dysregulated iron metabolism-related genes in TCGA-PAAD cohort. The expression levels of 67 differentially expressed genes (DEGs) related to iron metabolism are shown by a heatmap **(A)** and a volcano plot **(B)**. Venn diagram of 1969 DEGs and 527 iron metabolism-related genes **(C)**. Enriched Gene Ontology terms **(D)** and KEGG pathways **(E)** associated with the 67 DEGs.

Clinical pathological parameters of PAAD patients in TCGA database were shown in Supplementary Table S1. There were 56494 DEGs between the tumor tissues and the normal adjacent tissues, among them 1969 genes were screened with a threshold of *p* < 0.05 and an absolute log2-fold change > 1 (Figures 2A, B). After intersecting them with the 527 iron metabolism-related genes, we obtained 67 iron metabolism-related genes (including 22 up-regulated genes and 45 down-regulated genes) for subsequent analysis (Figure 2C). We explored the activities of chosen genes that were highly enriched for iron binding, heme binding, and tetrapyrrole binding using enrichment analysis (Figure 2D). The iron metabolism-related genes were mostly implicated in retinol metabolism pathways, chemical carcinogenesis, and arachidonic and metabolism pathways, according to pathway enrichment analysis (Figure 2E).

### Construction and assessment of the risk-score system

**FIGURE 3:**
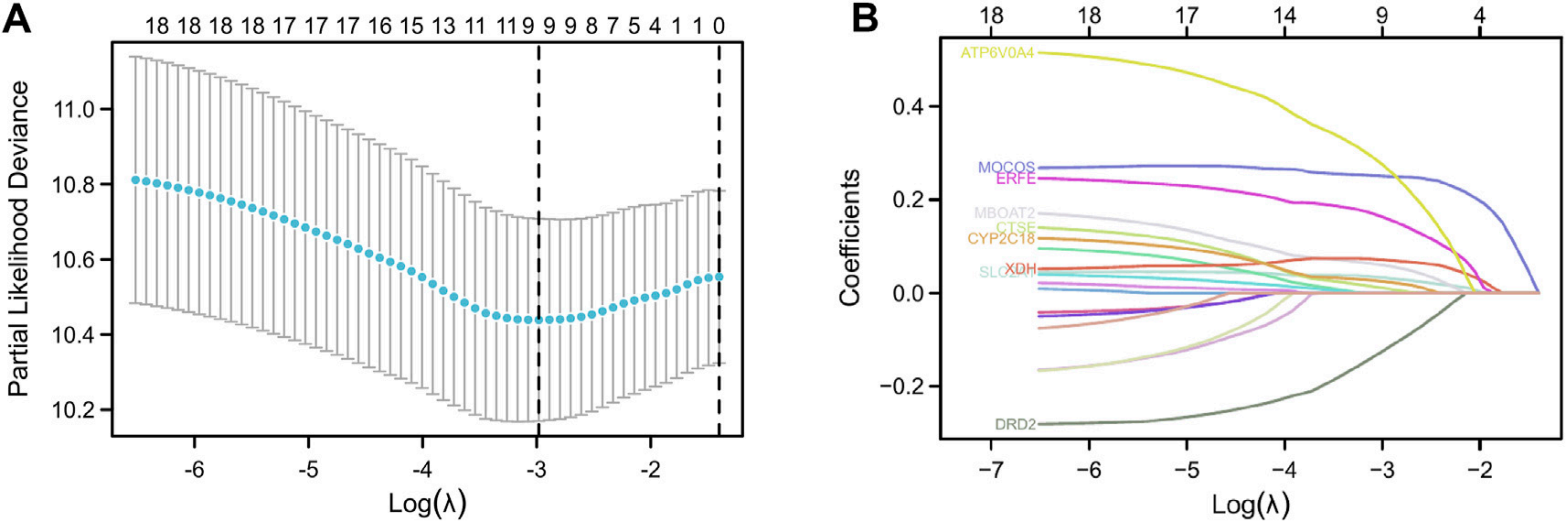
Construction of LASSO regression model. Cross-validation was used to tune parameter screening **(A)**, and coefficient profiles were shown in **(B)**.

The patients in the TCGA-PAAD dataset were split into two groups: training set (88 cases) and testing set (89 cases). Then, in the training set, we explored the relationship between the gene expression levels and OS time. With the Cox *p* < 0.05 criterion, 19 genes were defined as possible OS-related risk variables (Supplementary Table S1). Furtherly, 9 genes were finally screened by calculating the regression coefficients of the LASSO regression algorithm (Figures 3A, B; Supplementary Table S2). As a result, a 9-gene risk-score system was developed on the basis of the aforementioned formula.

**FIGURE 4:**
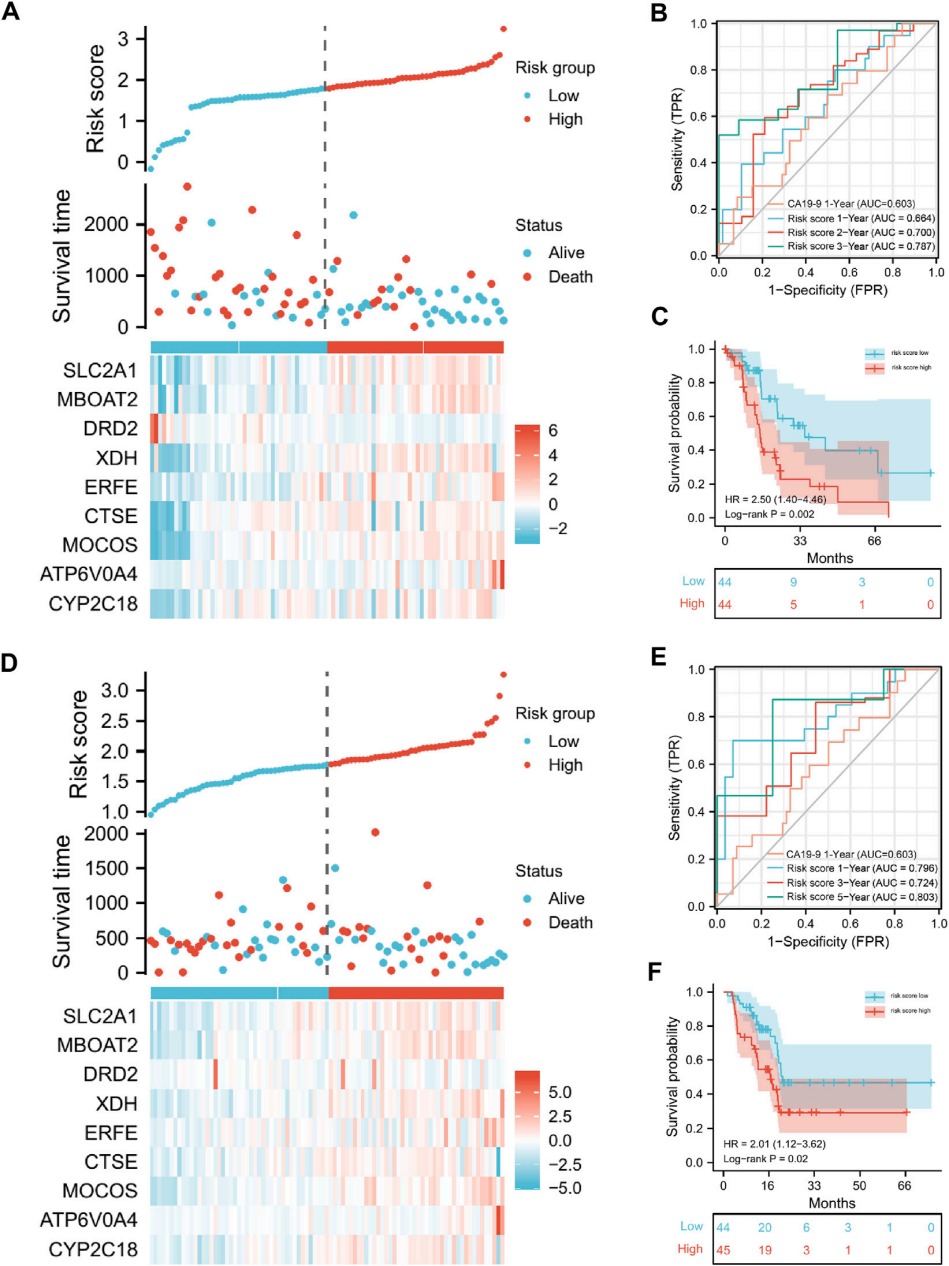
The presentation of the risk-score model in terms of risk score analysis, survival analysis and prognostic performance, in training and validation set. Risk score distributions and heatmaps of gene-expression levels in the training set **(A)** and validation set **(D)**. The risk score model’s ROC curves and AUC values for forecasting the 1-, 2-, and 3-year OS times in the training sets **(B)** and test sets **(E)**. In the training set **(C)** and test set **(F)**, Kaplan–Meier survival analysis was used to assess the OS times between the high- and low-risk groups.

Then, we calculated the risk score for each patient in the training and test sets separately. According to the median value of the risk score, patients with PAAD were separated into high- and low-risk categories. The heatmaps (Figures 4A, D) showed the 9 gene expression in different patients. In the training set, survival analysis revealed that the high-risk group’s OS rates were considerably lower than the low-risk group’s (*p* = 0.002, HR = 2.50; Figure 4C). AUCs at 0.664, 0.700, and 0.787, the ROC curves offered survival forecasts for 1, 2, and 3-year OS, correspondingly (Figure 4B). The predictive performance of risk score for one-year survival rate (average AUC 0.73) was higher than that based on CA19-9 (average AUC 0.603), a commonly used diagnostic indicator for PAAD.

Moreover, to verify the robustness of our approach, we used the test set for validation. Similarly, the AUCs for prognosis at 1-, 2-, and 3-year were 0.796, 0.687, and 0.724, respectively (Figure 4E), and the Kaplan-Meier analysis revealed that the patients in the high-risk group had considerably lower OS rates than those in the low-risk group (*p* = 0.02, HR = 2.01, Figure 4F). Together, these results indicated that our risk score model for predicting PAAD patients’ prognosis was of great robustness.

### The prognostic value and biological characteristics of screening genes

**FIGURE 5:**
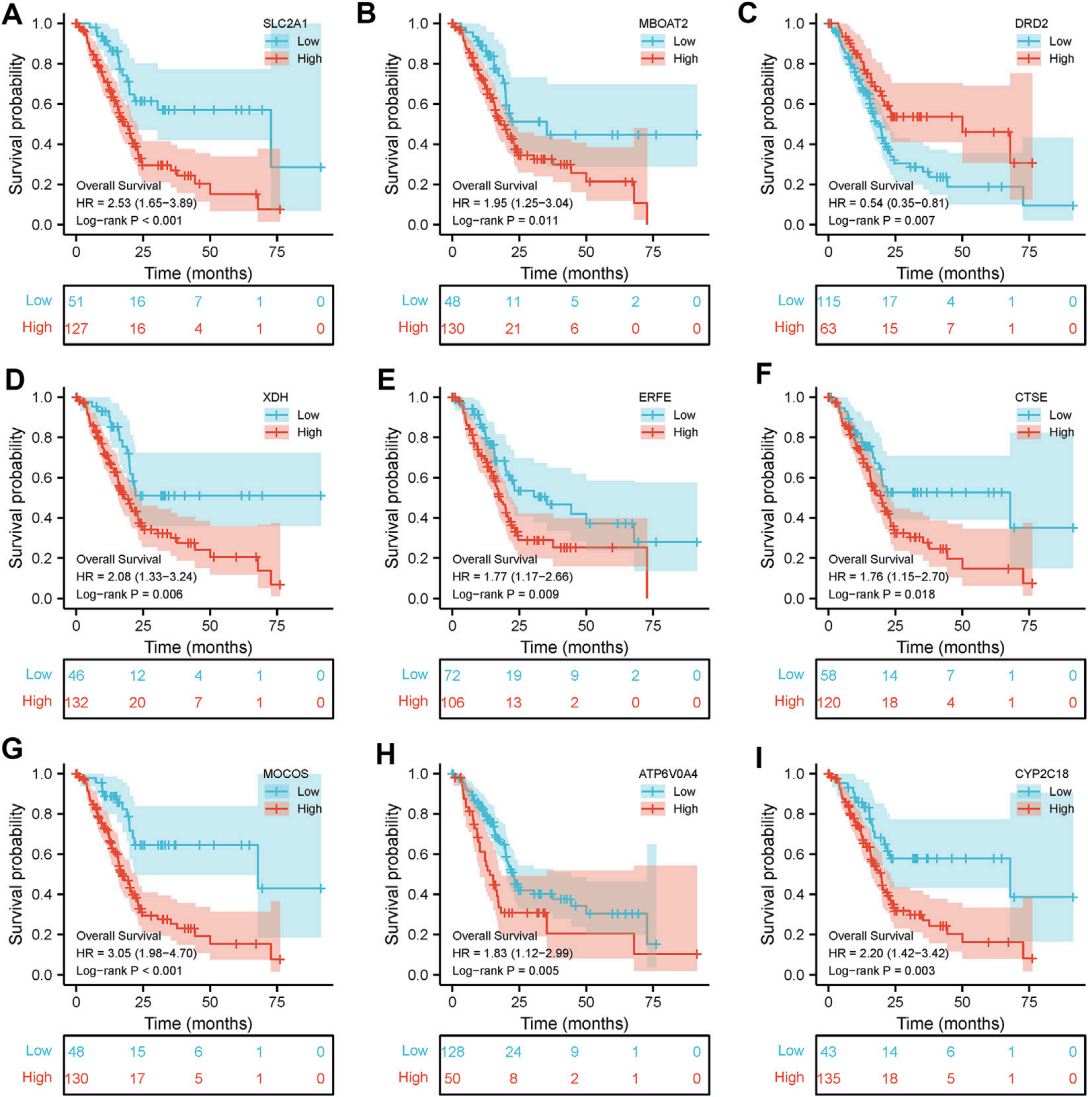
Kaplan–Meier survival analysis was performed on the 9 most valuable predictive genes.

**FIGURE 6:**
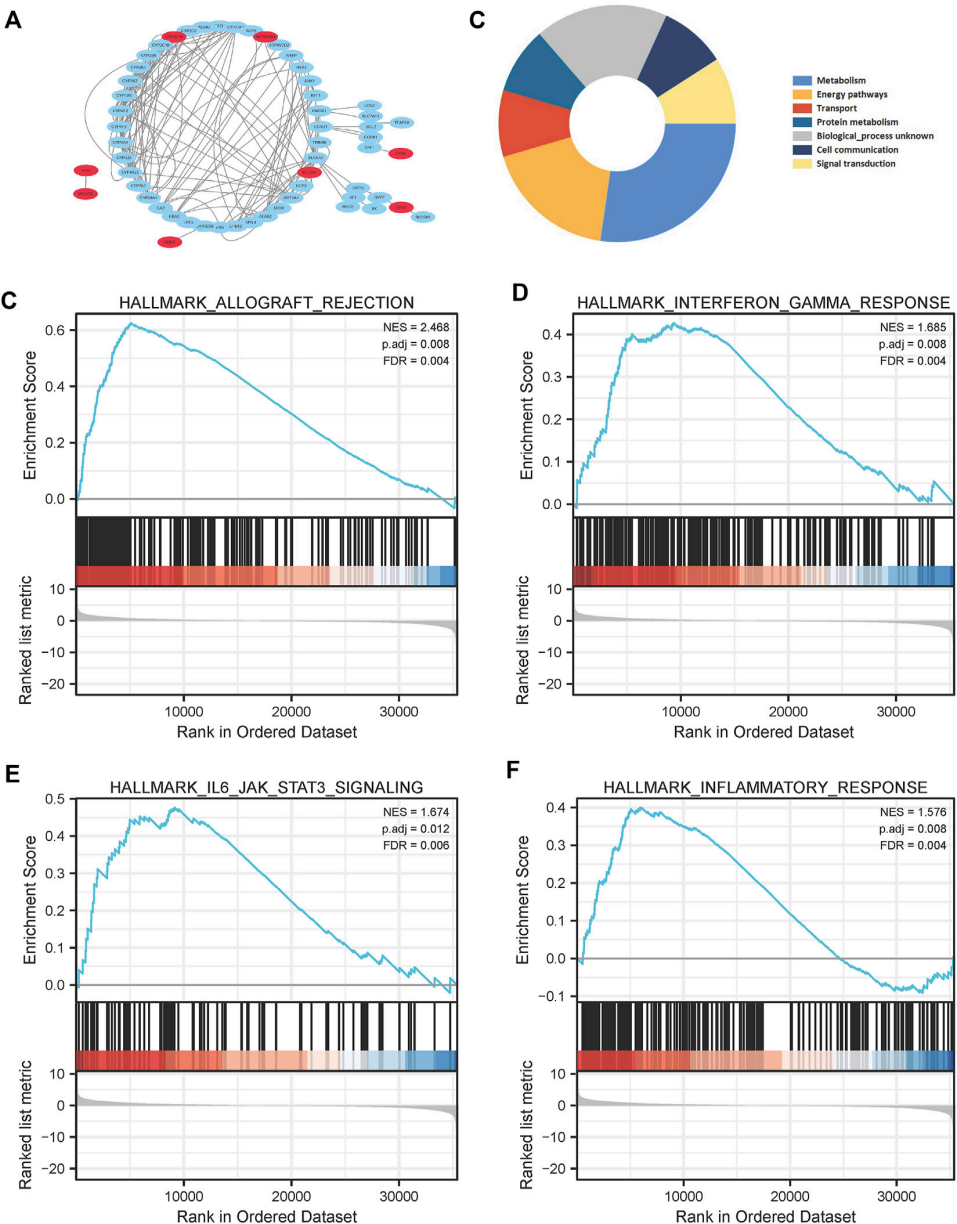
PPI network construction **(A)**, and Biological Process **(B)** of the 9 most valuable predictive genes. In the TCGA cohort, GSEA of the iron metabolism-related gene signature **(C–F)**, allograft rejection, interferon gamma response, IL6/JAK/STAT3 signaling pathway and inflammatory response were enriched in the high-risk group.

Results of Kaplan–Meier survival analysis indicated that nine iron metabolism-related genes significantly affected the prognosis of PAAD (*P* all <.05, Figure 5). The results demonstrated that higher expression of *SLC2A1*, *MBOAT2*, *XDH*, ERFE, *CTSE*, *MOCOS*, *ATP6V0A4*, *CYP2C18* and low *DRD2* expression were associated with worse prognosis. The PPI network showed extensive interactions between the nine iron metabolism-related genes and other proteins (Figure 6A). The findings of BP analysis revealed that these 9 iron metabolism-related genes were related to the biological process of metabolism, energy pathways, and transport (Figure 6B).

To determine the possible influence of the expression levels of iron metabolism-related genes on the PAAD transcriptome profile, GSEA analysis was performed comparing the high-risk and low-risk groups. Several pathways were found to be enriched in the high-risk group, including allograft rejection, interferon gamma response, IL6/JAK/STAT3 signaling pathway, and inflammatory response (Figures 6C–F).

**FIGURE 7:**
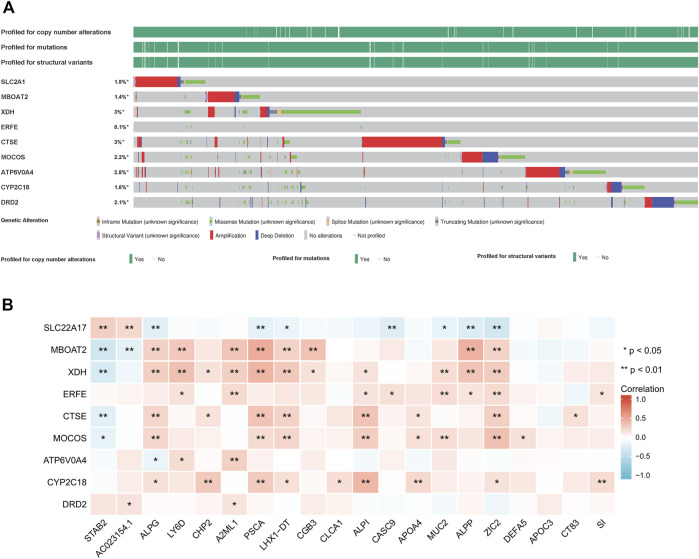
Mutation status of 9 iron metabolism-related genes **(A)** and their interaction with top 20 differentially expressed genes from tumor tissues of PAAD patient vs normal pancreatic tissue **(B)**.

A survey of 9 iron metabolism-related genes mutants across all cancer types were explored *via* cBioPortal database (Figure 7A). Among them, the gene with the highest mutation rate were *XDH* and *CTSE*, accounting for 3%, and the primary type of *CTSE* mutation was amplification, while the primary type of *XDH* mutation was missense mutation. In PAAD, the most genetic alterations were mainly in *MOCOS* and *CTSE*. *MOCOS* was altered in 7/184 (3.8%) cases, including 1.63% (3 cases) of amplification and 2.17% (4 cases) of deep deletion, *CTSE* was altered in 5/184 (2.72%) cases, including 1.09% (2 cases) of mutation and 1.63% (3 cases) of amplification. The low mutation rate may relate to the small PAAD sample size, and further validation of mutational burden as a predictive biomarker is necessary. In PAAD, mutations rs587784395, rs145069780, and rs200352240, which located in the genes of *SLC2A1*, *CTSE*, and *DRD2* respectively, were associated with the OS and prognosis period. Correlation between the screened genes and top 20 DEGs was shown in Figure 7B. Among them, *XDH* was positively correlated with *ALPG, LY6D, CHP2, A2ML1, PSCA, LHX1-DT, CGB3, ALPI, MUC2, ALPP, ZIC2*, and negatively correlated with *STAB2*.

**FIGURE 8:**
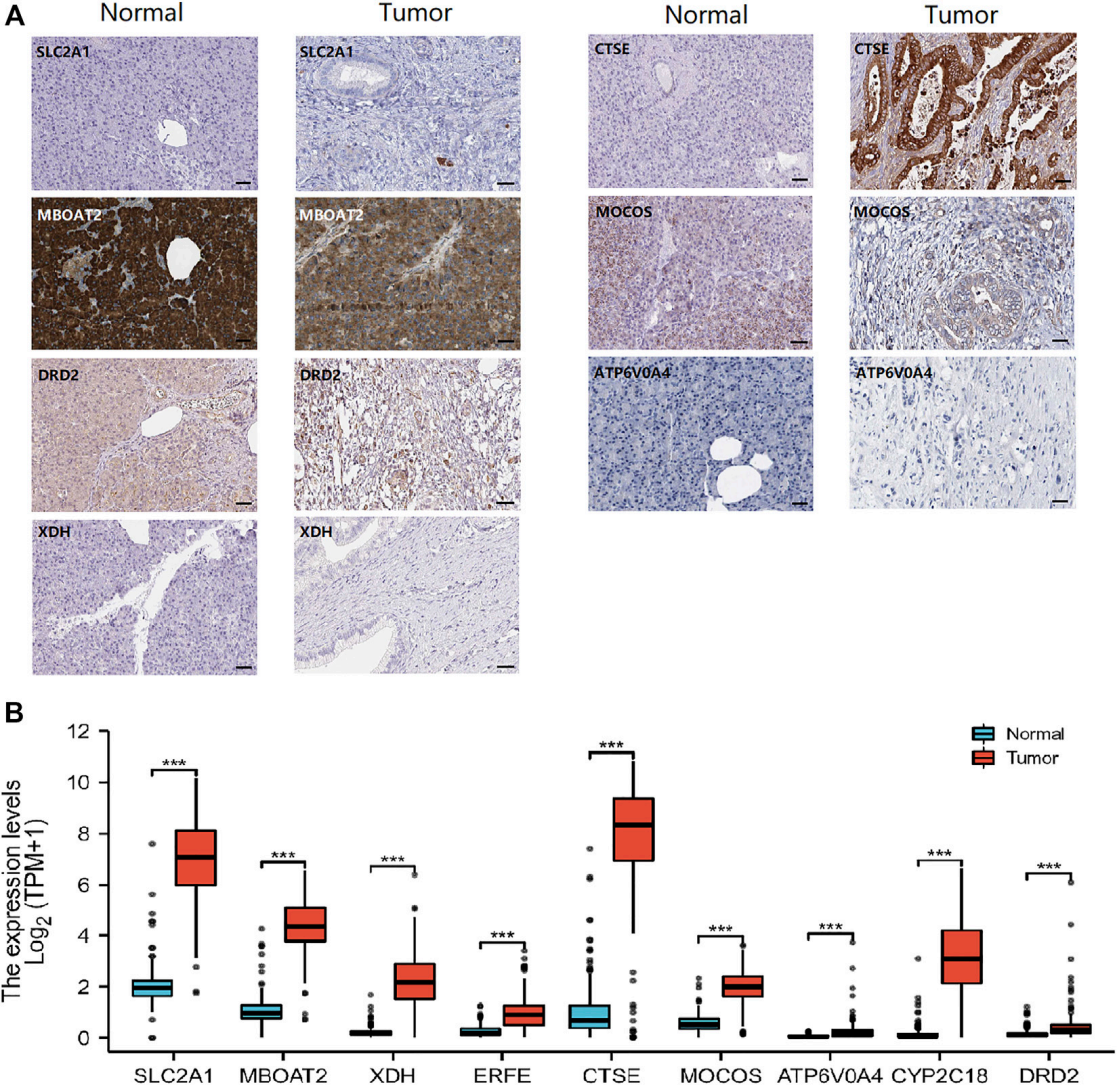
Human Protein Atlas (HPA) immunohistochemical images of PAAD and normal pancreatic tissue **(A)**, and transcriptomic data analysis of the nine screened genes between normal and tumor in HPA **(B)**. *p < .05, **p < .01, ***p < .001.

In addition, the HPA database, providing RNA-sequencing and immunohistochemical in PAAD and normal tissues, was performed to verify the transcript level and protein level of nine iron metabolism-related genes (Figure 8). Notably, normal pancreatic exocrine glandular cells and exocrine glandular cells stain positive for MBOAT2, while tumor tissue is highly positive predominantly in tumor cells (Figure 8A). The immunohistochemistry pictures of *ERFE* and *CYP2C18*, on the other hand, were not detected. In comparison of PAAD group with normal group, we found that the 9 iron metabolism-related genes were significantly up-regulated (*p* all < 0.01, Figure 8B), the trends of these genes were similar to the former result.

### Relationship between risk score and clinical characteristics

**FIGURE 9:**
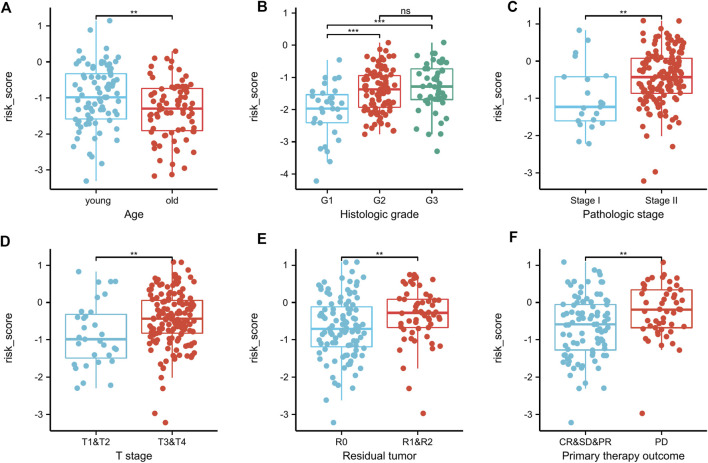
Correlation between clinicopathologic features and the risk score in the TCGA dataset **(A–F)**. In PAAD patients stratified by age, histologic grade, pathologic stage, T stage, residual tumor, and primary therapy outcome **(A–F)**, risk-score distributions revealed statistically significant differences. ^**^ *p* < .01, ^***^ *p* < .001, ns, not significant.

In this study, we considered the relationship for both the risk score and the clinical characteristics as well. In PAAD patients stratified by age, histologic grade, pathologic stage, T stage, residual tumor and primary therapy outcomes, risk-score dispersion revealed statistically significant variations (Figures 9A–F).

### Nomogram construction and validation

**FIGURE 10:**
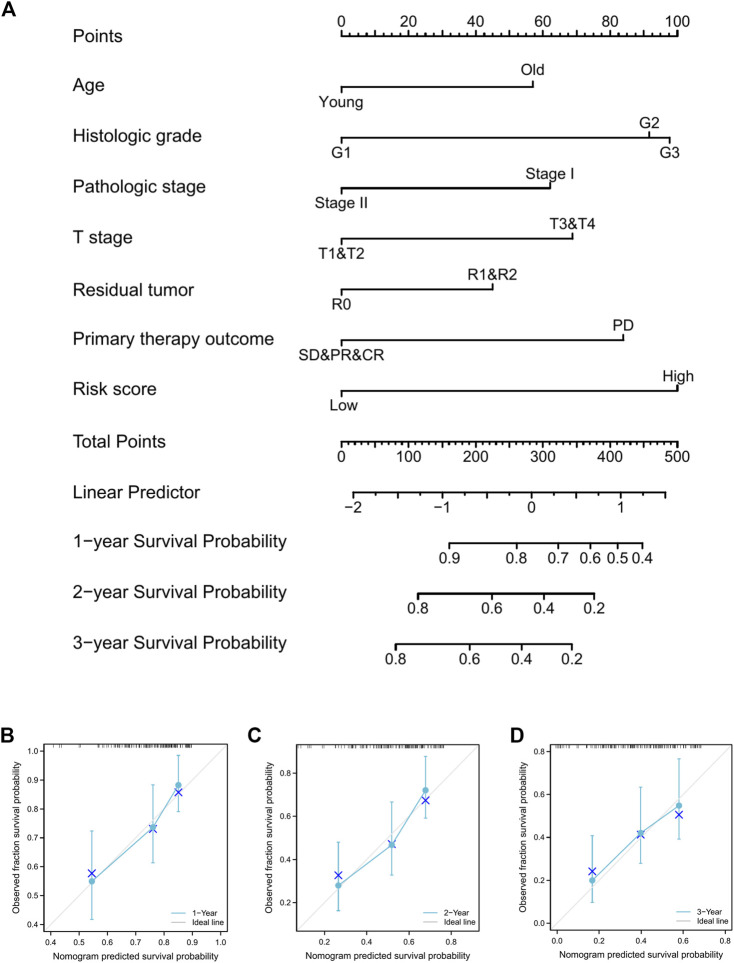
Prognostic nomogram for PAAD patients’ 1-, 2-, and 3-year OS times. **(A)**, Independent risk variables identified in the TCGA cohort *via* multivariate Cox regression were included into the nomogram model. The nomogram’s calibration curves for predicting 1-, 2-, and 3-year OS in TCGA-PAAD cohorts **(B–D)**.

Furthermore, a nomogram was constructed to predict the survival rate of PAAD patients at 1, 2, and 3 years. The nomogram comprised age, histologic grade, pathologic stage, T stage, residual tumor, primary therapy outcome, and risk score (Figure 10A). The results indicated that as contrasted to the low-risk group, the high-risk group’s OS rates were much lower (HR = 4.211, 95% CI = 2.466-7.193, *p* < .001; Supplementary Table S3). According to the calibration plots (Figures 10B–D), the nomogram was well calibrated, with average forecasted probability at one, two, 3 years OS rates concordant with actual probabilities.

### Quantitative PCR verification of results

**FIGURE 11:**
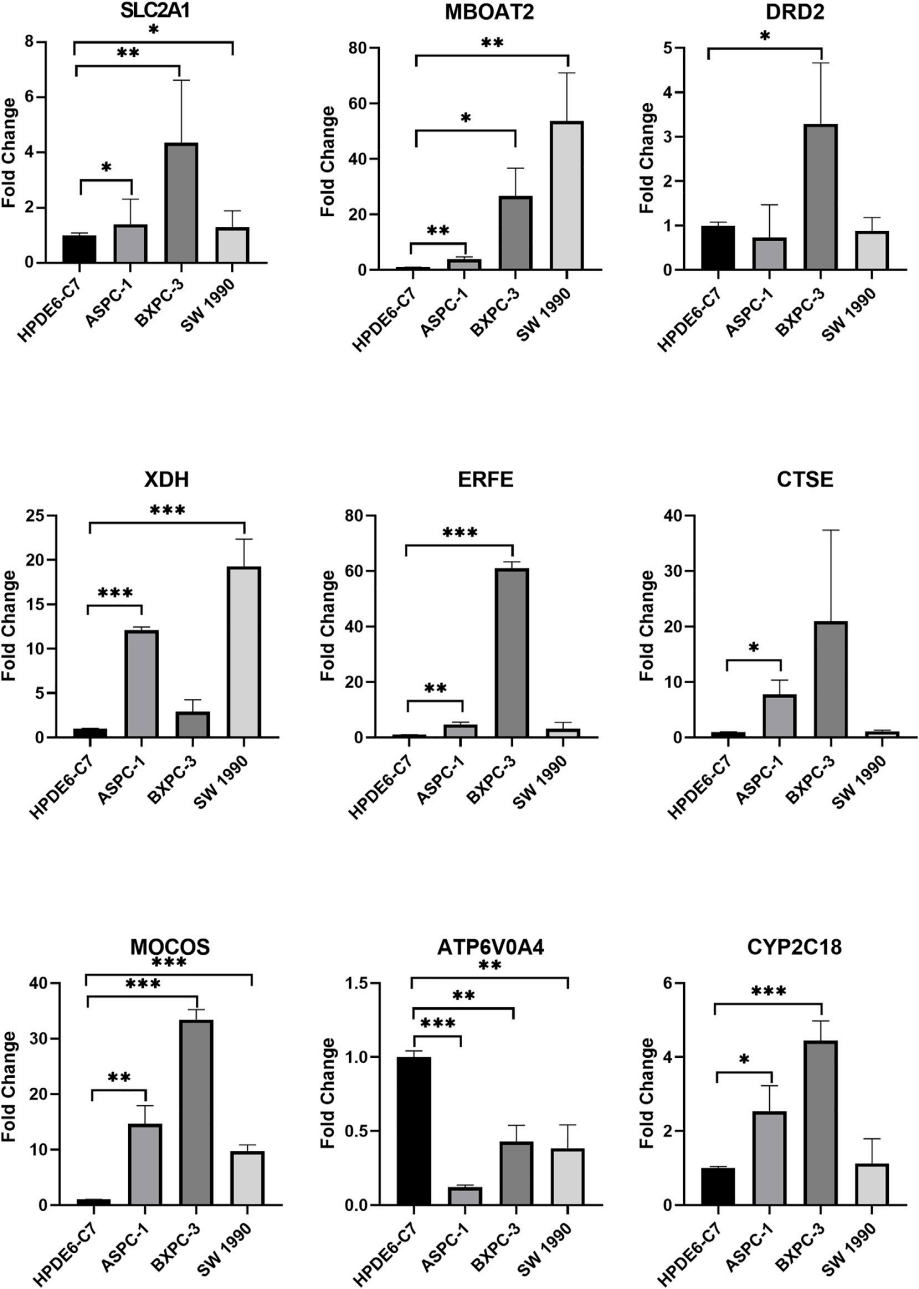
Quantitative PCR verification of the screened genes. *p < .05, **p < .01, ***p < .001.

These results were validated *via* qPCR analysis using three different cell lines, ASPC-1, BXPC-3, and SW 1990 (Figure 11). The result showed that *SLC2A1*, *MBOAT2*, and *MOCOS* were significantly up-regulated in the three PAAD cell lines (*P* all <.05), while *ATP6V0A4* was significantly down-regulated in the three PAAD cell lines (*p* < .05). *DRD2* was up-regulated in BXPC-3 (*p* < .05); *XDH* was up-regulated in ASPC-1 and SW 1990 (*P* all <.05); *ERFE* was up-regulated in ASPC-1 and BXPC-3 (*P* all <.05); *CTSE* was up-regulated in ASPC-1 (*p* < .05); and *CYP2C18* was up-regulated in ASPC-1 and BXPC-3 (*P* all <.05).

## Discussion

Pancreas is clearly involved in maintaining iron homeostasis throughout the body. The resultant systemic iron overload causes extremely high iron deposition in the liver, pancreas, and heart, and among other organs. There were a large number of literatures precisely indicated that iron absorption was generally increased in exocrine pancreatic dysfunction and chronic pancreatitis, thus demonstrating a possible connection between the iron regulatory pathway and the exocrine pancreas (Nicolas et al., 2001). Maintaining iron homeostasis may be beneficial to protect the health of the pancreas.

For this investigation, we utilized gene expression data as well as clinical-pathological information. First, 67 DEGs related to iron metabolism were screened. Then 9 genes were selected through univariate Cox analysis and LASSO regression analysis, which were identified as potential prognostic markers, and were then utilized to build a prognostic model. Among the selected genes, the expression levels of eight genes (*SLC2A1*, *MBOAT2*, *XDH*, *ERFE*, *CTSE*, *MOCOS*, *ATP6V0A4*, and *CYP2C18*) positively correlated with OS, whereas the expression levels of only one gene, *DRD2*, negatively correlated with OS. By using multivariate Cox regression analysis, we were able to confirm that the model we built was effective and stable in diverse patient cohorts, as well as an independent predictive marker. Although the genes that are potentially involved in the iron response and their respective contributions to PAAD are still unknown, our findings highlight the complexity of iron-associated metabolic pathways in allograft rejection, interferon gamma response, IL6/JAK/STAT3 signaling pathway, and inflammatory response. High iron contributes to allograft -mediated rejection is the conclusion that new treatments to lower allograft iron could be clinically impactful. Bioinformatic analysis places the interactions of interferon gamma responsiveness and iron metabolism into overlapping networks. Elevated iron indices have been associated with a poor response to interferon therapy (Sartorii et al., 2010). It has been confirmed that iron is also essential in the activation of STAT3 by IL6 in cell lines and tumors (Xue et al., 2016). Notably, iron homeostasis is also central in inflammatory responses, whereby NF-κB, TNF and NLR are known to be important regulators and have been implicated in cancer-related inflammation (Chan et al., 2018).

Iron metabolism-related genes are known to have a role in the pathogenesis of pancreatic cancer. *SLC2A1*, also known as glucose transporter 1 (*GLUT1*), is the main glucose transporter in somatic cells. Many malignant tumors, including pancreatic cancer, breast cancer, and prostate cancer have up-regulated *SLC2A1*, and the level of *SLC2A1* is closely related to the clinical stage, degree of differentiation and lymph node metastasis of pancreatic cancer (Liu et al., 2012). *MBOAT2*, also known as lysophosphatidylcholine acyltransferases (*LPCATs*), is related to the incidence and progression of a number of tumors. *MBOAT2* overexpression in pancreatic ductal adenocarcinoma (PDAC) has been related to a poor prognosis (Idichi et al., 2018). In several malignancies, including pancreatic cancer, *XDH*, a rate-limiting enzyme that catalyzes the last stage of purine metabolism, was a reliable predictor for poor prognosis in many cancers including pancreatic cancer. (Saidak et al., 2018). *CTSE* was found to be a potential early biomarker of PDAC (Pontious et al., 2019). *CYP2C18* belongs to the cytochrome P450 2C subfamily, which has a strong risk of cancer susceptibility (Agundez, 2004). Although *CYP2C18* was highly correlated with OS and upregulated in PAAD cell lines *via* qPCR, research on *CYP2C18* was mostly concentrated in the gastrointestinal tract and liver, with relatively few studies in pancreatic cancer and lack of immunohistochemistry image in the HPA database. Chen et al. detected *DRD2* expression in pancreatic islets *via* western blotting and dual fluorescence localization. (Chen et al., 2014). Pathak et al. reported a case of prolactinoma with liver metastasis of pancreatic polypeptide tumor, after taking the *DRD2* agonist, the serum pancreatic polypeptide level decreased to one-seventh of the original level, and liver metastases were significantly reduced. (Pathak et al., 2004). In this study, the *DRD2* gene was highly expressed in the TCGA-PAAD database (*vs*. normal tissue, *p* < 0.001). The results of q-PCR validation showed that the *DRD2* gene was significantly highly expressed in the BXPC-3 cell line (*vs.* HPDE6-C7 cell line, *p* < .05), but had no significant difference between the ASPC-1 and SW1990 cell lines (*vs.* HPDE6-C7 cell line, *p* < .001). BXPC-3, ASPC-1 and SW1990 are immortalized cell lines from patients with PAAD, the inconsistency results in different cell lines reflects the heterogeneity of DRD2. Other genes, such as *ERFE*, *MOCOS*, and *ATP6V0A4*, may be involved in tumorigenesis, metabolism, or treatment. (Wada et al., 2014; Papanikolaou and Pantopoulos, 2017; Park et al., 2020). Generally, gene expression was consistent with expression at the protein level, in this study, we collected RNA-sequencing data and immunohistochemical images in PAAD and normal tissues from HPA database. The observed discordance of transcript and protein levels is likely to be explained by regulation of translation, post-translation modifications, and protein turnover. However, whether the expression differences at the transcriptional level of these genes are consistent with the protein level remains to be further explored.

In this study, we established an iron metabolism-related polygene risk-score system for predicting prognostic of PAAD. The nomogram template contained risk scores and other clinical indicators. As evidenced by calibration plots and ROC curves, the nomogram offers a solid prediction capability for the OS rates of the PAAD, and indicated that the system we have constructed was reliable and effective, which could be used to determine the prognosis of patients and arrange follow-up plans. However, there are potential limitations to our study. First, the prognostic model is built using the TCGA database, although gene expression validation was performed in three PAAD cell lines, protein-level and animal-level functional validation was still lacking. In the next step, for the screened genes, the differential expression verification at the protein level can be carried out, and the cellular and animal function phenotypes can also be implement to provide more systematic functional validation. Second, due to the limited sample size, large-scale prospective surveys remain necessary to validate our risk-score system in the future.

## Conclusion

To conclude, we developed and validated a risk-score system for prognosis and risk stratification according to genes associated to iron metabolism. A nomogram model for predictions of OS rate over 1-, 2-, 3- year was built and demonstrated high predictive precision. The screened genes have the potential to be targets for exploring mechanisms related to iron metabolism in PAAD.

## Data Availability

The datasets presented in this study can be found in online repositories. The names of the repository/repositories and accession number(s) can be found in the article/Supplementary Material.
