# Supplementary material for: Development and validation of a prognostic prediction model for iron metabolism-related genes in patients with pancreatic adenocarcinoma
Source: Front Genet. 2023 Jan 4;13:1058062. doi: 10.3389/fgene.2022.1058062 (PMC9846079; doi:10.3389/fgene.2022.1058062)
Supplement: Supplementary file 3 [file Table1.DOCX]

**TABLE 1.** Clinical pathological parameters of PAAD patients

| Clinical pathological parameters | *N* | % |
| --- | --- | --- |
| Age |  |  |
| <=65 | 93 | 52.2 |
| >65 | 85 | 47.8 |
| Histologic grade |  |  |
| G1 | 31 | 17.8 |
| G2 | 95 | 54.6 |
| G3 | 48 | 27.6 |
| Pathologic stage |  |  |
| Stage I | 21 | 12.6 |
| Stage II | 146 | 87.4 |
| T stage |  |  |
| T1 | 7 | 4.0 |
| T2 | 24 | 13.6 |
| T3 | 142 | 80.7 |
| T4 | 3 | 1.7 |
| Residual tumor |  |  |
| R0 | 107 | 65.2 |
| R1 | 52 | 31.8 |
| R2 | 5 | 3.0 |
| Primary therapy outcome |  |  |
| SD | 9 | 6.5 |
| PR | 10 | 7.2 |
| CR | 71 | 51.1 |
| PD | 49 | 35.2 |

**TABLE 2. Iron metabolism-related genes and their relationship with OS, and their coefficients in LASSO regression model.**

| **Gene** | **HR (95%CI)** | ***P* value** | **Lasso_coef** |
| --- | --- | --- | --- |
| SLC2A1 | 1.243 (1.090-1.418) | 0.001 | 0.0329 |
| MBOAT2 | 1.414 (1.136-1.759) | 0.002 | 0.0598 |
| DRD2 | 0.631 (0.421-0.947) | 0.026 | -0.1222 |
| XDH | 1.307 (1.125-1.519) | <0.001 | 0.0714 |
| ERFE | 1.686 (1.243-2.285) | <0.001 | 0.1621 |
| CTSE | 1.150 (1.049-1.261) | 0.003 | 0.0108 |
| MOCOS | 1.735 (1.320-2.281) | <0.001 | 0.2506 |
| ATP6V0A4 | 1.621 (1.212-2.169) | 0.001 | 0.2725 |
| CYP2C18 | 1.221 (1.077-1.384) | 0.002 | 0.0248 |

HR, Hazard Ratio; 95%CI, 95% confidence interval.

**TABLE 3. Univariate and multivariate Cox analysis of OS in PAAD patients.**

| **Parameters** |  | **Univariate Cox analysis** | |  | **Multivariate Cox analysis** | |
| --- | --- | --- | --- | --- | --- | --- |
|  |  | **HR (95% CI)** | ***P*-value** |  | **HR (95% CI)** | ***P*-value** |
| Age | <=65 | - | - |  | - | - |
|  | >65 | 1.290  (0.854-1.948) | 0.227 |  |  |  |
| Histologic grade | G1 | - | - |  | - | - |
|  | G2 | 1.950  (1.003-3.791) | 0.049 |  | 1.602  (0.718-3.574) | 0.250 |
|  | G3 | 2.616  (1.299-5.266) | 0.007 |  | 2.091  (0.895-4.889) | 0.089 |
| Pathologic stage | Stage I | - | - |  | - | - |
|  | Stage II | 2.325  (1.065-5.073) | 0.034 |  | 1.537  (0.425-5.559) | 0.512 |
| T stage | T1&T2 | - | - |  | - | - |
|  | T3&T4 | 2.023  (1.072-3.816) | 0.030 |  | 1.516  (0.545-4.216) | 0.426 |
| Residual tumor | R0 | - | - |  | - | - |
|  | R1&R2 | 1.645  (1.056-2.561) | 0.028 |  | 1.399  (0.830-2.356) | 0.207 |
| Primary therapy outcome | SD&PR&CR | - | - |  | - | - |
|  | PD | 2.487  (1.570-3.940) | <0.001 |  | 1.980  (1.189-3.299) | 0.009 |
| Risk score level | Low | - | - |  | - | - |
|  | High | 4.461  (2.653-7.504) | <0.001 |  | 4.211  (2.466-7.193) | <0.001 |

HR, Hazard Ratio; 95%CI, 95% confidence interval.
