# Supplementary material for: Development and validation of a prognostic prediction model for iron metabolism-related genes in patients with pancreatic adenocarcinoma
Source: Front Genet. 2023 Jan 4;13:1058062. doi: 10.3389/fgene.2022.1058062 (PMC9846079; doi:10.3389/fgene.2022.1058062)
Supplement: Supplementary file 4 [file DataSheet1.ZIP › supplementary table.docx]

| **Gene** | **HR (95%CI)** | ***P* value** |
| --- | --- | --- |
| MOCOS | 1.735 (1.320-2.281) | <0.001 |
| FA2H | 1.248 (1.077-1.446) | 0.003 |
| ATP6V0A4 | 1.621 (1.212-2.169) | 0.001 |
| CYP2C18 | 1.221 (1.077-1.384) | 0.002 |
| CCND1 | 1.340 (1.097-1.638) | 0.004 |
| SLC2A1 | 1.243 (1.090-1.418) | 0.001 |
| CCNB1 | 1.555 (1.210-2.000) | <0.001 |
| CYP2J2 | 1.246 (1.012-1.535) | 0.039 |
| TFAP2A | 1.326 (1.103-1.595) | 0.003 |
| MBOAT2 | 1.414 (1.136-1.759) | 0.002 |
| LCN2 | 1.137 (1.037-1.247) | 0.006 |
| DRD2 | 0.631 (0.421-0.947) | 0.026 |
| XDH | 1.307 (1.125-1.519) | <0.001 |
| STEAP1 | 1.376 (1.125-1.683) | 0.002 |
| CYP2S1 | 1.134 (1.020-1.260) | 0.020 |
| ERFE | 1.686 (1.243-2.285) | <0.001 |
| TMPRSS6 | 0.770 (0.635-0.933) | 0.008 |
| AGMO | 1.215 (1.050-1.405) | 0.009 |
| CTSE | 1.150 (1.049-1.261) | 0.003 |

**Supplementary Table 1. 19 genes were defined as potential OS-related risk factors**

**Supplementary Table 2. Primer Sequences of RNAs for RT-qPCR**

| **RNAs** | **Forward Primer (5’-3’)** | **Reverse Primer (5’-3’)** |
| --- | --- | --- |
| SLC2A1 | GGCCAAGAGTGTGCTAAAGAA | ACAGCGTTGATGCCAGACAG |
| MBOAT2 | TTTTGCTTTGGATGGTATGCCT | GCACACTGTGAGGTATCCCA |
| DRD2 | CAACGGGTCAGACGGGAAG | GAATTTCCACTCACCTACCACC |
| XDH | CCAAATTGCTGCATGAACCAG | TGCTTCCGAGGAGTGTCTTTC |
| ERFE | CCGGAGCCAGGGTTGATTC | GCACTCCATGAGAACATGAAGAG |
| CTSE | AGGCATCCGTCCCTCAAGAA | CCTTGGCACTCTGGTCCATTG |
| MOCOS | GCAGGAACTGTCTATCTTGACC | TGCGCCAGGATTCTGTAGC |
| ATP6V0A4 | CTCCCACGGGAAATGATTACC | CGTCTCAAAGAAGTCTTGGGTT |
|  |  |  |
| CYP2C18 | GGAAAACGGATGTGTATGGGAG | GTGGCACACGACCAAATGC |
|  |  |  |
| GAPDH | TGACTTCAACAGCGACACCCA | CACCCTGTTGCTGTAGCCAAA |
